# One-step formation of three-dimensional macroporous bacterial sponges as a novel approach for the preparation of bioreactors for bioremediation and green treatment of water†

**DOI:** 10.1039/c8ra04219e

**Published:** 2018-09-03

**Authors:** Areej K. Al-Jwaid, Dmitriy Berillo, Irina N. Savina, Andrew B. Cundy, Jonathan L. Caplin

**Affiliations:** School of Environment and Technology, University of Brighton Brighton UK; Engineering Technical College/Basrah, Southern Technical University Basrah Iraq; School of Pharmacy and Biomolecular Sciences, University of Brighton Brighton UK i.n.savina@brighton.ac.uk; School of Ocean and Earth Science, University of Southampton Southampton UK

## Abstract

Immobilisation of bacteria on or into a polymer support is a common method for the utilisation of bacteria as biocatalysts for many biotechnological, medical and environmental applications. The main challenge in this approach is the time taken for the formation of stable biofilms, and the typically low percentage of bacterial cells present on or in the polymer matrix. In this work we propose a novel method for producing a porous bacteria based structure with the properties of a sponge (bacterial sponge) that we then use as a bioreactor for water treatment. Cryogelation has been used as a tool to create macroporous (*i.e.* with pores in the range 10–100 μm), highly permeable systems with low diffusion constraints and high bacterial content (more than 98% to total material content). A novel crosslinking system was used to form stable bacterial sponges with a high percentage of live bacteria organized in a 3D porous structure. The bacterial sponge was produced in a one step process and can be made from one or several bacterial strains (in this case, two bacterial strains *Pseudomonas mendocina* and *Rhodoccocus koreensis* (and a mixture of both) were used). Reduction of the total polymer content to 2% makes the system more sustainable and environmentally friendly under disposal as it can be simply composted. The bacterial sponges have good mechanical stability and cell viability, which enables repeated use of the materials for phenol degradation for up to five weeks. The material can be stored and transported in cryogenic conditions (−80 °C) for prolonged periods of time, retaining its bioremediation activity following 4–6 weeks of frozen storage. The proposed method of producing bioreactors with a high number of live immobilised bacteria, low polymer content and controlled 3D structure is a promising tool for developing novel materials based on active bacterial cells for various environmental, biotechnological, biological and medical applications.

## Abstract 2

A novel method of crosslinking live bacteria into a stable 3D porous structure and its subsequent use in phenol degradation is reported.

## Graphical abstract

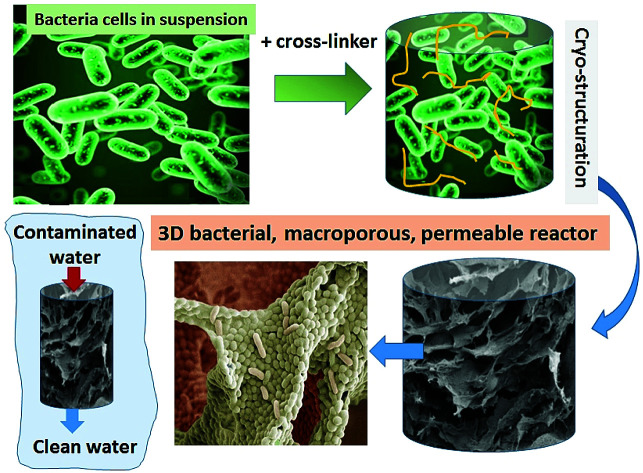

## 1. Introduction

Today, bacteria are utilised for many applications as one of the more cost-effective and eco-friendly approaches for dealing with modern industrial demands. Bacteria are used as biocatalysts and help to break down waste products, to synthesize medically important compounds (antibiotics, proteins), to produce fuel and electricity and much more. Bacterial immobilisation techniques are versatile and economic methods that are used in various industries to improve the biotechnological process. Immobilisation allows easy separation of the cells from the product and their reuse in subsequent process steps, making the whole process more economically feasible and efficient. Immobilising microbial biomass is also an important step in enhancing and increasing efficiency for substrate conversion. This is due to the physical isolation of bacterial cells from the external medium, minimization of cell loss during the technological process and creation of a favourable environment protecting cells from harsh ‘’external’’ conditions (pH, high contaminant concentrations, *etc.*).^1–7^ These benefits have encouraged the use of microbial immobilisation systems in a number of green chemistry applications in different fields, including the medical, biological and biotechnological sectors.^8–11^

**Fig. 1:**
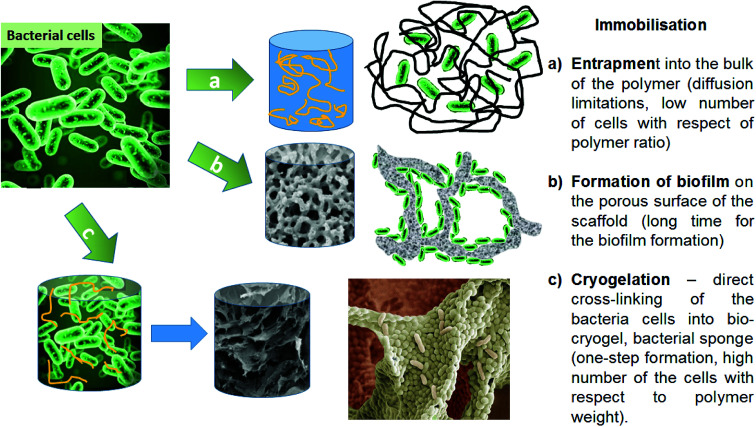
Strategies for bacterial cell immobilisation onto scaffold/polymer supports, and their benefits and limitations.

Bacterial cells can be immobilised by imbedding into a polymer matrix, or by developing a biofilm on a suitable surface or porous support (Fig. 1).^12^ The immobilisation of bacterial cells into cryogel materials is considered a robust approach for biotechnological applications.^13–15^ Cryogels have several desirable features compared to other macroporous hydrogels.^16^ They possess high mechanical strength during twisting, elongation or squeezing, which can reduce the damage both to the cryogel scaffold and to its inherent macroporous structure during application in physically aggressive treatment settings. Additional benefits include their synthesis using non-toxic water-soluble components, and their relatively simple production at moderately low cryogelation temperatures and defrosting at room temperature.^17^ These features make them ideal carriers or scaffolds to hold and protect microbial cells, to enable them to function as catalysts in various biotechnological and environmental applications.

The use of crosslinking agents is another strategy that can be used to immobilise bacterial cells. The concept of crosslinking of bacterial cells is based on chemical reactions between amino groups on the bacterial cell surface and aldehyde groups from the cross-linking polymer. These reactions can be used to produce macroporous crosslinked cell cryogels as monolithic structures *via* a cryogelation process.^18^ Producing bio-cryogels, based on crosslinking the bacterial cells with very low concentrations of polymers, is a novel approach in biotechnological applications.^14,19^

Here, for the first time, we use a facile one-step cryogelation procedure to produce 3D macroporous bacterial constructs (bio-cryogels, or bacterial sponges) based on immobilized *Pseudomonas mendocina* and *Rhodococcus koreensis* (23%) with less than 1% of polymeric cross-linker, and apply these for degradation of phenol. The mass ratio of bacteria to polymeric cross-linker was about 30 to 1, whereas in previously published work related to entrapped bacterial cells in PVA cryogel walls this ratio was 1 to 60–66.^20^ The mechanical and biological stability of the bio-cryogel was assessed *via* microscopy, rheological analysis and cell viability assays. *Pseudomonas* spp. and *Rhodococcus* spp., along with *Acinetobacter* spp. and *Bacillus* spp., have been confirmed to utilise phenol as a source of energy. Phenol and its derivatives are USEPA priority pollutants and are commonly found in untreated industrial effluents. Previously, bacteria for bioremediation of phenol were immobilized by formation of a biofilm on to a polyethylene oxide cryogel surface, with the cryogel preparation and the following biofilm formation completed in 9 days, and requiring the use of sophisticated equipment such as UV lamps (400 W).^21^ The main aim of this paper is to demonstrate the one-step preparation of stable units of novel macroporous cryogel materials (as 3D “bioreactors”) using mainly bacteria with a minimum concentration of crosslinking polymer, and demonstrate their potential application as water treatment tools.

## 2. Materials and methods

### 2.1. Materials

Two strains of known phenol-degrading bacteria, *Pseudomonas mendocina* (*P. mendocina*) (NCIMB 13264) and *Rhodococcus koreensis* (*Rh. koreensis*) (NCIMB 13709) were supplied by the National Collection of Industrial, Food and Marine Bacteria (NCIMB), U.K. Tryptone soya broth (TSB), tryptone soya agar (TSA) and phosphate buffered saline (PBS) were purchased from Oxoid Ltd, U.K. Phenol, sodium hydrogen carbonate, sodium chloride, glycerol, ammonium chloride, thiazolyl blue tetrazolium bromide (MTT, 98%), Live/Dead Bac Light kit (catalogue number: L7007) were purchased from Fisher Scientific Co., UK. Other chemicals, including 4-aminoantipyrine, glutaraldehyde, polyvinyl alcohol (PVA) (86.7–88.7%) (*M*_w_ 67 000), polyethyleneimine, linear (*M*_w_ 423), Pur-A-Lyzer TM Mega 1000 Dialysis Kit (1 kDa and 14 kDa pore size) were obtained from Sigma-Aldrich (U.K.). Potassium ferricyanide and ammonium hydroxide were purchased from Acros Organics (U.K.).

### 2.2. Activation of bacterial strains and culture conditions

Lyophilised cultures of *P. mendocina* and *Rh. koreensis* were reconstituted following instructions provided by the NCIMB. Under aseptic conditions, each lyophilised strain was suspended in 0.5 mL of (TSB) in 1.5 mL vials and a loop of cells cultured onto TSA plates and incubated at 30 °C for 24 hours. Broth cultures of each strain were prepared by adding sterile glycerol as a cryo-protectant at a concentration of 5% (v/v) to 1 mL of each culture. This was transferred into 1.5 mL vials and kept at – 80 °C for future use.

### 2.3. Synthesis of aldehyde-modified polymers

Different types of cross-linker solutions were prepared. Polyethyleneimine-aldehyde solution (PEI-al) was prepared by mixing 7.65 mL of 2% polyethyleneimine (PEI) in distilled water (neutralised to pH 7.5 by adding 2.0 M hydrochloric acid) with 9.38 mL of 10% glutaraldehyde (GA, added drop wise). The solution was stirred at room temperature for 2 h, and an orange colour in the obtained solution indicated the formation of Schiff's bases. The solution was then immediately transferred into a Spectropore dialysis bag with 1000 Da pore size and dialysed against 5 L of distilled water for 3 days, changing the water twice a day. Unreacted GA in the dialyzed water, due to its potential toxicity, was oxidised to glutaric acid by potassium permanganate prior to disposal. The presence of aldehyde groups in the wash solution was estimated using a reaction with fuchsine.^22^ The obtained PEI-al was sterilised *via* filtering through a 0.45 μm membrane and stored in the refrigerator until future use. Polyvinyl alcohol-aldehyde (PVA-al) was modified as previously described elsewhere.^15^ Briefly, a polyvinyl alcohol (PVA) solution was prepared by dissolving 0.6 g of PVA powder (67 kDa, 99% degree of deacetylation) in 10 mL of warm distilled water with stirring and heating at 80–85 °C until complete dissolution. Then, the PVA solution was allowed to cool before adding 0.3 mL of 50% glutaraldehyde solution at room temperature. After one-hour incubation with stirring the unreacted GA was removed by dialysis against 5 L distilled water for three days. The presence of aldehyde groups in the PVA-al and PEI-al was confirmed using FTIR and ^1^H-NMR analysis (Fig. S1, S2 and S3†).

A group of multiples in the range of 3.55–3.70 ppm in the spectra (Fig. S1a†) corresponds to backbone protons of PEI, which shifted after the modification of the polymer. The condensation reaction of the primary and secondary amino groups of PEI with GA leads to the disappearance of amine functionality at 2.6 ppm (Fig. S1b†). The backbone protons of GA moiety appeared at 1.25–1.75 ppm and in the field of 5 ppm attributed to the hemiacetal form of GA (Fig. S1d†).^23^ Aldehyde groups were observed at 9.6 ppm (Fig. S1a†). The proton of Schiff's base in the ^1^H-NMR spectrum of PEI-al appeared at 8.36 ppm.^24–26^ PVA-al has two additional signals at 7.7 and 6.3 ppm which are attributed to the presence of protons of the cyclic acetal form and multiples at 2.23 ppm related to glutaraldehyde moieties (CH_2_(4H)) and the singlets at 2.1 ppm attributed to an ethyl groups (CH_3_) of PVA (Fig. S1c†). Considering the relation of integrals of peaks for backbone –(CH_2_–CH(R)) *n*-of PVA at 1.6 ppm and the characteristic signal of glutaraldehyde (CH_2_(4H)), one can conclude that the ratio between structural units of PVA to GA was 14.8 : 1 (Fig. S1c†). FTIR spectra of PEI-al and PVA-al showed carbonyl groups at 1714 cm^−1^ and 1717 cm^−1^, respectively, which indicated the presence of free aldehyde groups in the polymer structure.

### 2.4. Preparation of bio-cryogels with immobilised (crosslinked) *P. mendocina* and *Rh. koreensis*

Initially, *P. mendocina* and *Rh. koreensis* were grown in TSB culture to determine a growth curve for each strain and an estimation time for each phase. A single colony from each species was inoculated into individual tubes containing 10 mL of TSB and incubated at 30 °C for 24 hours to obtain fresh active cells. One mL of each culture was transferred into 500 mL of TSB and incubated again on an orbital incubator at 150 rpm and 30 °C until the stationary phase was reached. Pelleted bacterial cells were harvested *via* centrifuging broth cultures for 10 minutes at 10 000 rpm and 4 °C. All supernatants were discarded, and the pelleted cells were washed with 20 mL of ice-cold 0.9% sodium chloride solution and then used to prepare the crosslinked cell cryogels (labelled CCC), which had the following bacterial cells – cross-linker mass ratio (wet mass/dry mass of polymer): CCC1 60 : 1; CCC2 20 : 1; CCC3 24 : 1; CCC4 27 : 1. The final concentration of bacteria in the suspension was kept constant (at 23%). CCC1, CCC2, CCC3, CCC4 contained 0.385% GA; 0.385% GA & 0.77% PVA; 0.77% PVA-al & 0.19% PEI-al; and 0.385% PVA-al &0.46% PEI-al, respectively.

The CCC samples were produced by mixing 0.15 g of pelleted cells of each bacterial strain with 0.5 mL of each cross-linker solution to form a suspended solution of cells and polymers. This was transferred immediately into 9 mm diameter glass tubes closed with a rubber stopper at the bottom and frozen at −12 °C for at least 3 days in a Julabo cryostat (model F34 G13, Germany). After cryogelation CCC samples were thawed at room temperature and washed with sterile distilled water to remove non-crosslinked cells, and subsequently organized into three groups to estimate their ability to degrade phenol. These comprised (i) fresh samples of *P. mendocina*, (ii) samples of a 1 : 1 mixture of *P. mendocina* and *Rh. koreensis* frozen at −80 °C for four weeks (iii) samples of *Rh. koreensis* frozen at −80 °C for six weeks. For long term storage of the material, the cryogels were placed into 25 mL of carbonate buffer supplemented with 5% of glycerol solution and frozen immediately by liquid nitrogen and stored at (−80 °C). Samples were incubated with TSB for 24 hours, washed with distilled water and then incubated in 40 mL of carbonate buffer containing phenol at a concentration 50 mg L^−1^.

### 2.5. Material characterisation

The morphologies of the CCC samples were examined using scanning electronic microscopy (SEM). Samples were cut into thin slices (2 mm thick) using a sterile scalpel and washed with sterile (PBS). Samples were fixed by adding 5% glutaraldehyde solution overnight which was replaced with a fresh sterilised solution of PBS and changed three times before soaking in fresh sterilised deionised water for one hour twice. Then, the CCC samples were frozen at −80 °C for three hours before placing them into a Christ ALPHA 2–4 freeze-dryer for 24 hours. For SEM images, each freeze-dried sample was coated with a layer of platinum using a Quorum (Q150TES) coater and scanned using a Zeiss Sigma field emission gun SEM (Zeiss NTS).

The rheological properties of the CCC samples were assessed using a rheometer (ThermoHaake, type, 379-0001, Germany). Following thawing of the samples at room temperature, samples were cut into approximately 2 mm thick slices with a sterile scalpel for subsequent rheological testing. Their mechanical properties (dynamic storage modulus G′ and dynamic loss modulus G′′) were assessed. Conditions such as temperature, amplitude stress and frequency, were set to sinusoidal oscillating stress with small amplitude for identifying the linear viscoelastic region for all samples of CCC for both individual bacterial strains and their mixture. Then, an oscillating frequency sweep test was set up over a range of oscillation frequencies (0.1–50 Hz) at constant temperature (25 °C) and with an oscillation amplitude of 0.1–50 Pa, respectively, to obtain more information about the structural behaviour of the CCC samples.

The viability of crosslinked bacterial cryogel samples was determined by using the LIVE/DEAD® bacterial viability kit (catalogue number: L7007) and confocal laser scanning microscopy (CLSM, Leica TCS SP5). All samples of the different CCC were cut into slices approximately 1 mm thick with sterile scalpels and washed with distilled water to remove non-crosslinked cells. The samples were washed twice with 0.9% NaCl buffer and stained with SYTO 9 stain (wavelength 480/500 nm) and propidium iodide (wavelength 490/635 nm) for 15 minutes at room temperature under dark conditions according to the protocol of the LIVE/DEAD® Bacterial viability kit.

The concentration of live cells was also assessed by MTT (tetrazolium salt) assay according to the protocol of.^27^ The absorbance dye of MTT was measured at 595 nm wavelength using KC Junior software and a microplate reader (BioTeck Instruments). The concentration of live immobilized bacteria in the cryogel or in suspension after freezing was calculated by applying a calibration curve between the counted number of standard culturing of cell suspension on TSA using the colony forming unit (CFU) procedure, and the OD595 measurement of the same suspension dilutions of bacterial cells incubated with MTT for 90 minutes.

### 2.6. Preparation of free bacterial suspensions and 3D bioreactors for phenol and chlorophenol degradation

Free bacterial suspensions and CCC were tested for their ability to degrade phenol and 4-chlorophenol in batch cultures. Pelleted cells were prepared and used as free cells while CCC made previously were thawed at room temperature and washed with distilled water after incubation in 10 mL of TSB for 24 hours. Both free and crosslinked cells samples were incubated in 40 mL of 50 mg L^−1^ phenol or 4-chlorophenol solution with 25 mM carbonate buffer at pH 7.0 ± 0.5. Controls comprised phenol or 4-chlorophenol plus buffer only, and cross-linking polymer only without cells in phenol and buffer.

### 2.7. Determination of phenol concentrations

Phenol and 4-chlorophenol concentration was determined according to methods described previously^28,29^. A 100 μL aliquot of each incubated sample was diluted to 1 mL with distilled water, and 50 μL ammonium buffer was added to adjust the pH to 10.0 ± 0.3. This was followed by adding 25 μL of 4-aminoantipyrine solution and 25 μL of potassium ferricyanide. Reddish brown coloured solutions were produced because of the reaction with phenol. The solutions were filtered using 0.22 μm syringe filters and measured at a wavelength 510 nm.

The final concentration of phenol and 4-chlorophenol and its possible derivatives in water were also estimated using HPLC. A reverse phase C18 column was used with washes of 1% methanol & acetic acid and 1% aqueous acetic acid, 75/25, at a flow rate of 1 mL min^−1^ with a UV detector (275 nm). The injection volume of the sample was 40 μL in 50% methanol. Sample preparation included the dilution of the solution in pure methanol, freezing it at −20 °C for several hours, its centrifugation to remove proteins and filtering through a 0.22 μm filter to remove mechanical impurities and other unwanted compounds affecting the column. The retention times for the phenol and 4-chlorophenol were 5.5–5.6 min and 11.0 min, respectively.

## 3. Results and discussion

### 3.1. Preparation and stability of bio-cryogels

**Fig. 2:**
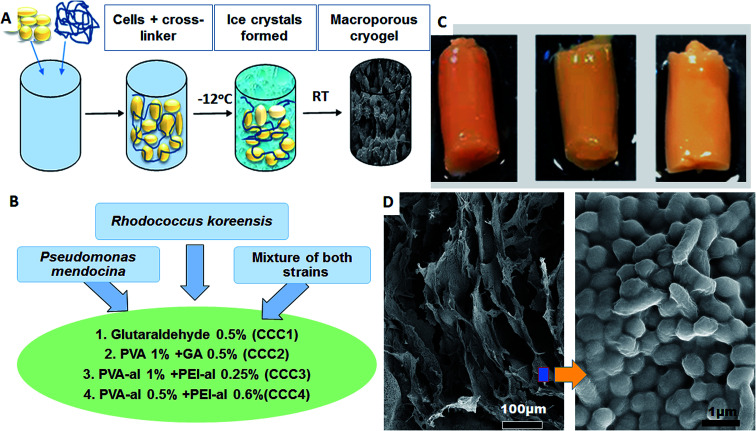
(A) Scheme of cryogel preparation, (B) types of bacterial strains and four cross linker polymer solutions used, (C) CCC4 of *P. mendocina* (left), *Rh. koreensis* (middle) and mixed cells (right), (D) SEM images of *P. mendocina* cross-linked in CCC4.

*P. mendocina* and *Rh. koreensis* in combination with four cross-linking agents were used as the main materials to form a monolithic macroporous structure of cross-linked cells *via* the cryogelation process (Fig. 2). The bacterial cells were crosslinked with a combination of 4 crosslinking systems (Fig. 2B). The combination and concentration of the cross-linkers was prior optimized to achieve the most stable construct. Bacteria were crosslinked based on the reactions between aldehyde groups of the cross-linking polymer and amino groups that are found on the surface of the cells.^15^ Cryogelation was used to help to shape the crosslinked bacteria into the porous monolith/bacteria sponge. During the cryogenic treatment, *i.e.* freezing at a temperature below the freezing point of the solvent, the bacterial cells and polymer come into close contact as they are expelled by the forming ice crystals, and the reaction between the functional groups on the polymer and bacterial cell membrane occurs in the frozen state (Fig. 2A). After defrosting large voids and micro channels are formed in place of the ice crystals. Scanning electronic microscopy (SEM) images of CCC samples of *P. mendocina*, *Rh. koreensis* and their mixture (1 : 1) demonstrated the macroporous structure of these materials, with cells closely attached to each other forming a 3D structure with well-developed channels as a result of the cryo-structuration process (Fig. 2D). The material contains up to 95% water, most of which is free water and could be easily removed by squeezing, thus the whole material performs as a sponge.

GA and PVA have been used previously to crosslink cells.^30,31^ However, the numbers of live bacteria following cross-linking/cryogelation were not investigated. In the present study particular attention was paid to maintaining the number of viable bacterial cells after immobilization as well as the stability of the 3D monolithic structure. A series of cross-linker systems were assessed and their composition and concentration was optimized to achieve high viability and mechanical strength. PVA was added to the system as a biocompatible, non-toxic polymer to decrease the toxicity effect of GA. Crosslinked cell cryogels produced from *P. mendocina* using GA (CCC1) and a combination of PVA and GA (CCC2) were structurally unstable and started to disintegrate from the first day of incubation with phenol and buffer until the crosslinked structure had completely disintegrated at the fifth day of incubation. These results disagree with earlier reports of production of a structurally stable cryogel using GA to crosslink two different bacterial strains *Caldicellulosiruptor saccharolyticus* and *Clostridium acetobutylicum*,^14,18^ which may be related to a difference in conditions, such as pH. It is known that the Schiff's base formed as a result of aldehyde interaction with amino-groups is not stable at acidic conditions; this may lead to accelerated decomposition of the material. Another reason could be that the aforementioned strains tend to form a biofilm during the cryo-structuration process which in turn enhances the mechanical properties of the structure.

A combination of PVA and PEI was also analysed. PEI has high solubility in water, and possesses large amounts of amino groups which make it widely used in biological applications.^32^ PVA and PEI were activated with glutaraldehyde to obtain activated polymers containing aldehyde groups (PVA-al and PEI-al)^14,15,19^ and their toxicity during the bio-cryogels formation was assessed. Cryogel based on only PVA-al has good elasticity, however poor water permeability, whereas the cryogel produced from bacteria and PEI-al had reasonable water permeability but has brittle mechanical properties. PVA-al and PEI-al were used together as a mixed polymer in various concentrations to determine the optimum ratio that could produce a stable monolithic construction of crosslinked cells with a high population of live bacteria and a high efficiency for phenol degradation. Ranges of CCC samples with various combinations of the two cross-linkers were prepared and two concentrations of each polymer solution were chosen as described in Fig. 2B, which gave an optimum stability, structure and preliminary ability to degrade phenol (data not shown).

**Fig. 3:**
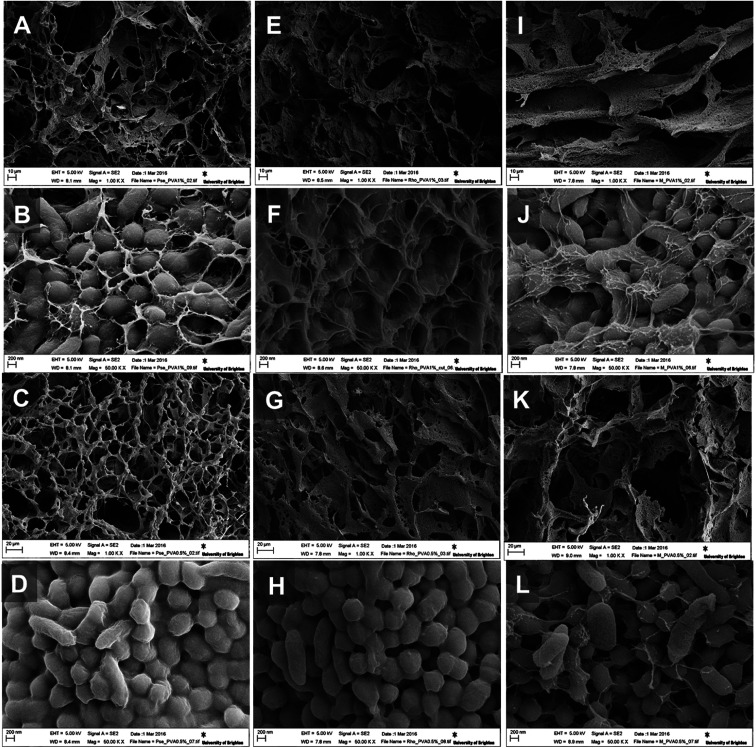
SEM images of *P. mendocina* (A–D), *Rh. koreensis* (E–H) and mixed cells (I–L) cryogels crosslinked with CCC3 (top images: A, B, E, F, I and J) and CCC4 (bottom images: C, G, K, D, H and L).

We were able to achieve a high concentration of cells (99%) keeping a minimum concentration of the polymer (1%) to achieve good structural integrity. As can be seen on SEM images for CCC3 samples, only a thin layer of crosslinking polymer covering the cells is visible (Fig. 3A, F and J) whereas, the CCC4 samples have even denser organization of cells with a decreased percentage of cross-linked polymer (mass ratio 27 : 1) on the cell surfaces (Fig. 3D, H and L). The cells are tightly packed into the thin bacterial film which forms the walls of the 3D structured monolith with well-developed channels filled with liquid (Fig. 3). The bacterial walls are very thin (about a one-three cell thickness), thus each cell has good access to the aqueous solution with non-limited mass-transport of nutrients and by-products. The macropores (10–100 μm) provide good permeability inside the monolith and there are no diffusion limitations. This kind of morphology is very important for several applications such as the use of such materials as bioreactors for synthesis of chemicals and proteins or for breaking down hazardous materials, and as biosensors or microbial fuel cells. The proposed method allows production of complex bacterial constructs from a one-step procedure, which opens the opportunity for developing completely novel materials. For all of these applications retaining the viability of cells is critically important.

Various factors may play important roles in the reduced viability of cells following freezing and cryogelation. The freezing conditions and ice crystal formation may damage the membrane of the cells, or there may be an absence of sufficient oxygen during the cryo-structuration process. Another important factor that could affect the CCC samples is the cross-linker itself. It is known that glutaraldehyde used as an individual cross-linker can easily penetrate the cell membrane and disrupt metabolic pathways even at low concentrations, resulting in cell death.^33^ PVA and PEI were selected as relatively non-toxic polymers that show no side effects on the metabolic activity of live cells.^14,34^ However, after the modification with GA this could change.

**Fig. 4:**
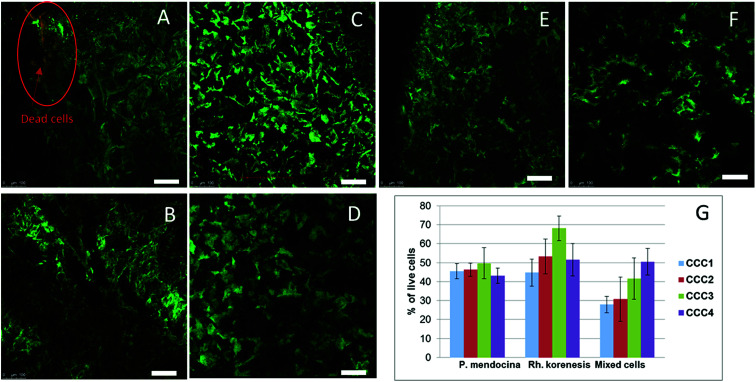
CLSM images of crosslinked cells cryogels stained with Live/Dead kit: *P. mendocina* (A and B), *Rh. koreensis* (C and D) and mixed cell (E and F) crosslinked with (CCC3) (A, C and E) and with (CCC4) (B, D and F) (scale bar is 100 μm). The percentage of live cells of *P. mendocina*, *Rh. koreensis* and their mixture estimated by MTT assay in bio-cryogel samples crosslinked with CCC1, CCC2, CCC3 and CCC4 (G), frozen free cells were taken as (100%).

The viability of cells in the cryogel structure after production was assessed using the Live/Dead Bac light kit.^35^Fig. 4(A–D) indicates that most of the bacteria were alive (stained green) after cross-linking and undergoing freeze–thawing conditions during the cryogelation process. There were a few dead bacteria, which appeared as red spots in the images.

The MTT assay showed lower cell viability in the bio-cryogels compared to frozen free (non-crosslinked) cells, as a consequence of the freezing conditions (such as the effect of growing ice crystals) damaging the cell structures (Fig. 4G). Samples cross-linked with (CCC1), (CCC2) and the combination of two activated polymers with various percentages (CCC3 and CCC4) showed viability percentages ranging from 29 to 68% compared with frozen free cells. Despite PVA and PEI being considered non-toxic the percentages of live cells in CCC produced from the modified polymers (PVA-al and PEI-al) were also reduced. This reduction did not significantly differ from that given by the GA and GA+PVA system (according to statistical analysis using an ANOVA test at the 0.05 level, as shown in Fig. 4G). There was no significant difference between *P. mendocina* and *Rh. koreensis*, however the mixture of both (1 : 1) showed a slightly lower number of live cells. Overall, about 40–50% of the cells used for the CCC formation remain viable and as will be shown below were able to maintain their metabolic activity (assessed by their ability to degrade phenol).

**Fig. 5:**
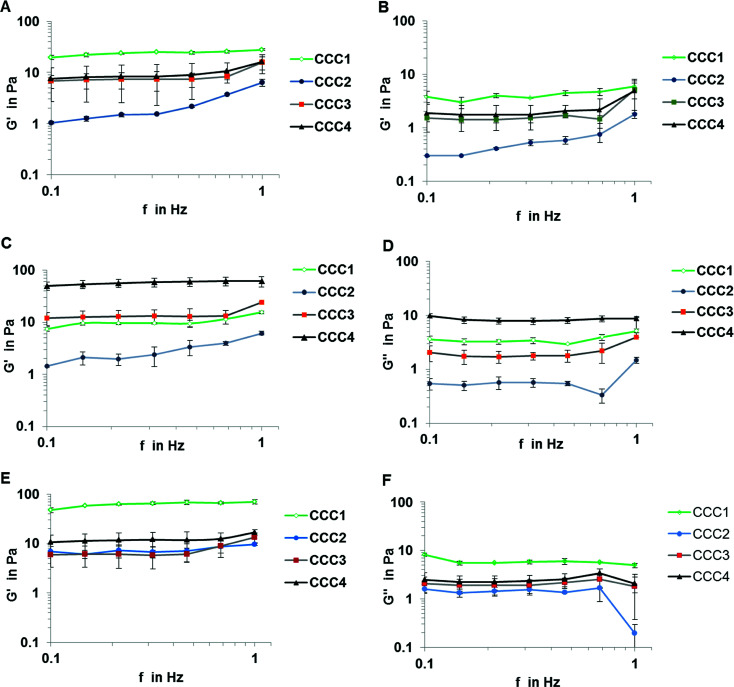
Rheology diagrams for all bacterial strains *P. mendocina* (A and B), *Rh. koreensis* (C and D) and mixed cultures (E and F) crosslinked with four cross-linked polymer solutions CCC1, CCC2, CCC3 and CCC4. (*n* = 3).

The mechanical properties of CCC samples are important in assessing their potential utility in industrial applications. Based on the rheological analysis the storage (elastic) modulus G′ and the loss (viscous) modulus G′′ showed a quasi-linear trend over frequencies between 0.1 and 1 Hz. The dominance of G′ over G′′ for all bacterial strains confirmed that all samples formed gelled structures. The value of the G′ and G′′ moduli for *P. mendocina* and mixed cells followed the order CCC1 > CCC2 > CCC3 > CCC4, (CCC1 showed highest values in of mixed strains), which could be related to higher packaging of cells due to shape difference (rods and spherical cell: cross-linker ratio 60 : 1), in contrast, CCC samples produced from *Rh. koreensis* using CCC3 and CCC4 cryogels showed better viscoelasticity compared with samples formed by conventional crosslinking polymers (CCC1 and CCC2) as shown in Fig. 5, which is most probably related to a larger linker spacer with higher flexibility and multipoint attachment due to the branched structure of PEI-al and PVA-al, whereas GA has only aldehyde groups providing single linking. The bio-surfactant materials that are produced by cells extracellularly or as part of the cell membrane are anionic, neutral or cationic (of which the last type contains the amino groups^31^). As *Rhodococcus* spp. is one of the cationic bacterial groups, the reaction between the aldehyde groups from the modified polymers and the amino groups on the surface of the cells was increased, which explains the higher viscoelasticity (G′) of samples prepared from *Rh. koreensis* and modified polymers (CCC3 and CCC4) compared with CCC1 and CCC2. *Rhodococcus* is characterized by a spherical structure which therefore can form a more compact structure compared to the randomly organized rod shaped *Pseudomonas*, which agrees with the viscoelasticity data (Fig. 5A and B).

Despite the CCC1 and CCC2 samples showing an initial gel structure, this was maintained for only two days before rapid disintegration, until the samples were destroyed completely on the fifth day of incubation, which may be related to gradual hydrolysis of Schiff's base groups over time. To examine whether the hydrogen bonding between the polymer and bacterial cells improves the mechanical properties of the bio-cryogels a combination of PVA forming physical gel and GA cross-linking agent was applied. The incorporation of PVA into the structure did not improve the storage (elastic) modulus of the material CCC2 of *P. mendocina* or *Rh. koreensis*, which is related to the larger volume of non-frozen liquid micro phase and therefore more diluted suspension of bacteria leading to less efficient cross-linking of bacteria, compared to CCC1 (Fig. 5A and C). The CCC3 and CCC4 samples crosslinked with modified cross-linking polymers showed much greater stability and reusability over the five weeks of the same experiment, due to the significant molecular length of the linker with a number of the functional groups and multipoint attachment to the cell membrane inhibiting rapid decomposition of the structure even if the hydrolysis of some Schiff's base groups takes place. G′ and G′′ for samples crosslinked with modified agents were also assessed after one-week incubation with phenol and carbonate buffer. The results show that samples produced from *Rh. koreensis* were reduced in elastic and viscous modulus by 11.75% and 24.49% for CCC3 and 51.7% and 54.58% for CCC4, respectively. *P. mendocina* and mixed cells samples showed a higher G′ value, and slightly lower G′′ value, compared with initial values for fresh prepared samples which is in agreement with^36^ who highlighted that the existence of layers of lipopolysaccharides on the outer surface of Gram negative cells increased as a part of their adaptation to the new environment and confirmed that bacterial strain types (Gram positive or Gram negative) may play an important role in mechanical structure characteristics.

Another factor that may affect the rheological properties of the bio-cryogel samples is concentration and molecular size of the cross-linking polymers. From Fig. 5 samples made of mixed bacterial strains showed higher values of G′ and G′′ compared with those from individual strains. The two different shapes of cells may influence the diffusion rate of the crosslinking polymers and effect the accessibility of the amino groups on the bacterial surface. It is easier for the large polymers to react with more amino groups on the surface of the cells.^37^ Furthermore, the concentration of the polymer solution can also affect the G′ value, as after converting the water to ice crystals, higher concentrations can promote greater intermolecular connections which then keep their structure more coherently on thawing.^38^ The storage (elastic) modulus G′ for CCC3 and CCC4 was comparable to cryogels prepared *via* self-assembly of 1% wt/v solution of Fmoc-Phe-Phe^39^ and kefiran^40^ and up to 0.5% of xanthan at (−20 °C). Therefore, the modified polymers containing more aldehyde groups and which were added as more concentrated solutions (*i.e.* which interacted most effectively during freezing conditions at (−12 °C)) showed higher G′ values, in contrast, further increments of cross-linker polymer concentration may reduce the G′ value according to.^41^

To examine the possibility of long-term storage of the prepared bio-cryogel samples, which would allow their production and storage and/or transport before use, bioremediation activity after prolonged storage at −80 °C was tested. The CCC samples of *Rh. koreensis* and (a 1 : 1 volume) mixture of *P. mendocina* and *Rh. koreensis* cells were frozen for four and six weeks, respectively, at −80 °C. CCC1 and CCC2 samples disintegrated after thawing at room temperature. CCC3 and CCC4 samples retained their structure after thawing. These results confirmed the robust structure of samples produced from the aldehyde-modified polymers after prolonged low-temperature storage and defrosting. In addition, their phenol degrading activity of CCC3 and CCC4 was maintained over five weeks (5 testing cycles) after thawing (see below).

### 3.2. Phenol degradation

**Fig. 6:**
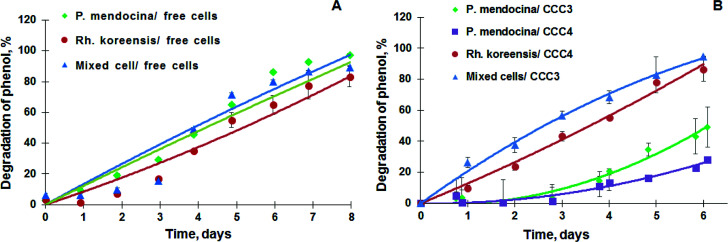
(A) Degradation of phenol by the suspension of *P. mendocina*, *Rh. koreensis* and their mixture (1 : 1). (*n* = 2), (B) degradation of phenol by *P. mendocina* CCC3 and CCC4 with a crosslinked number of cells equal to 5 × 10^8^, *Rh. koreensis* CCC4 and their mixture CCC3 (1 : 1) (*n* = 2).

*P. mendocina* and *Rh. koreensis* were selected as they both show an ability to degrade aromatic compounds.^42,43^ It was found that these two strains and their mixtures degrade phenol and require about a week to consume phenol completely (Fig. 6A) About five days were required for both strains to adapt to the new incubation conditions and start the exponential degradation phase. These results agree with the work of^44^ who showed that bacteria have a relatively long lag phase for adaptation. In Fig. 6A*P. mendocina* cells showed a slightly quicker adaptation compared to the individual strain of *Rh. koreensis* and mixed cells. The latter, however, showed a steeper exponential phase, probably because of competition between both species.^45^

**Fig. 7:**
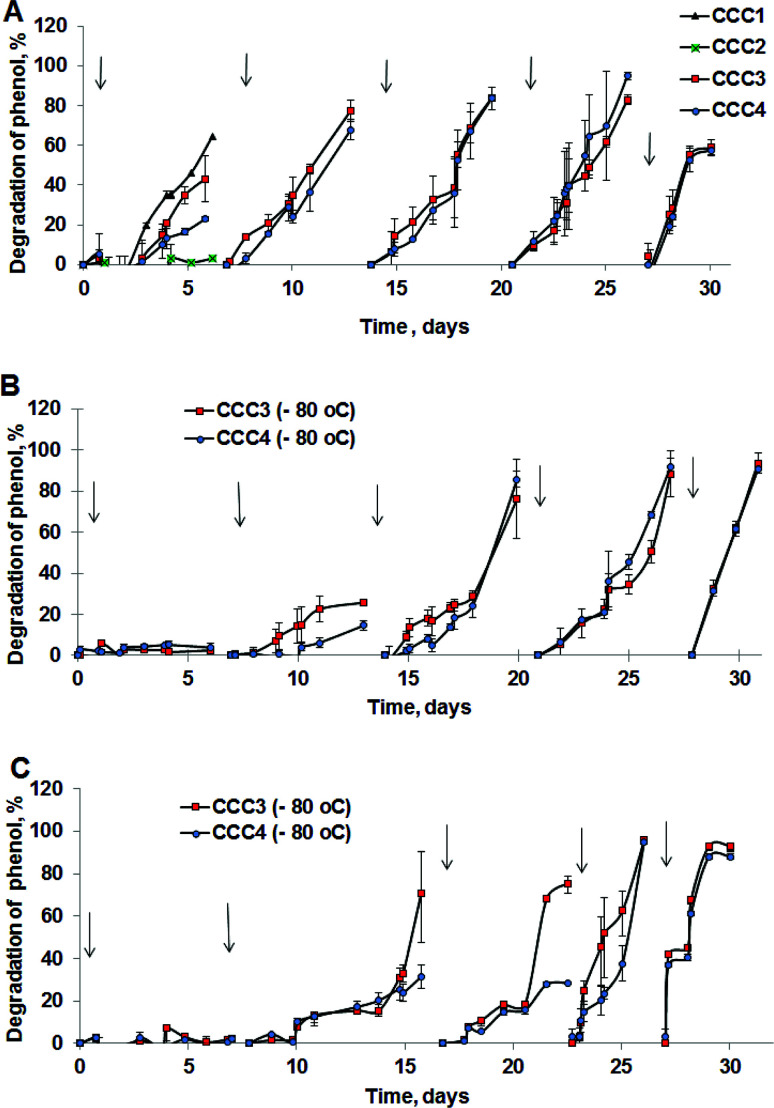
Degradation of phenol in repeat treatment cycles by (A) *P. mendocina* cross-linked with (CCC1), (CCC2), (CCC3) and (CCC4) with a crosslinked number of cells equal to 5 × 10^8^ for all samples (*n* = 2). (B) *Rh. koreensis* cross-linked with CCC3 and CCC4 stored in a frozen state with an initial number of cells (5 × 10^8^). (C) Mixed cells (*Rh. koreensis* and *P. mendocina*) stored in a frozen state at −80 °C (CCC3 with a few crosslinked cells = 2 × 10^7^ and CCC4 with number of crosslinked cells = 5 × 10^8^). *The arrows show a change of incubation media and addition of a fresh portion of phenol solution.

The degradation of phenol by CCC3 and CCC4 is presented in Fig. 6B. Cryogels crosslinked by GA (CCC1) or PVA+GA (CCC2) visibly disintegrated (*i.e.* naturally degraded) from the end of the first cycle and could not be reused as a monolith for subsequent cycles, thus, these samples were not considered for further investigation. All following experiments were done with CCC3 and CCC4 (combination of PVA-al + PEI-al), which retained their structural integrity and showed better performance, which continued for seven days, and these materials could be reused for several cycles (Fig. 7). A slower rate of phenol degradation was observed during the first cycle for *P. mendocina* CCC3 and CCC4 (Fig. 6) which gradually increased over the subsequent cycles, slightly dropping again in the fifth bioremediation cycle (Fig. 7A). The crosslinked mixture of *P. mendocina* and *Rh. koreensis* CCC3 was more active initially compared to other types, although this reversed over time. Overall, the stability of CCC3 and CCC4 based on [PVA-al 1% + PEI-al 0.25%] and [PVA-al 0.5% + PEI-al 0.6%], and the metabolic activity of the cells and their ability to degrade phenol, continued over five cycles of incubation (Fig. 7A). Notably, cross-linked cells showed faster adaptation to the phenol-spiked environment than free cells, although the samples were not activated in the culturing media after each cycle.

According to the amino antipyrine method, in samples at the end of the bioremediation cycle the absorbance was zero or slightly negative, indicating that only a trace amount of phenol was left after incubation over 6 days at 30 °C in the presence of carbonate buffer. The near-complete degradation of phenol was confirmed using HPLC analysis (at a detection limit of 0.05 mg L^−1^). Typical phenol by-products such as catechol & protocatechuate were not present in the treated media (Fig. S4a–c†). However, several peaks appeared with a retention time in the range of 1 & 2 minutes which most probably related to tri-carbonic acids (Fig. S4a–c†).

The efficiency of CCC to degrade phenol after storage under frozen conditions was also evaluated, to assess their ability to be stored after production, transported and then reactivated for use at the site of application (*e.g.* industrial facility, wastewater treatment works *etc.*). Samples of *Rh. koreensis* and the mixture of *P. mendocina* and *Rh. koreensis* (1 : 1) were thawed and tested for phenol degradation after storage them at −80 °C for prolonged periods (4 weeks or greater). As noted above, CCC1 and CCC2 samples did not maintain their structural integrity and disintegrated after thawing at room temperature.

Frozen CCC3 and CCC4 samples required one week to recover following prolonged storage under frozen conditions and start their phenol-degrading activity (Fig. 7B and C). The phenol degradation rate gradually increased during the following weeks. Adaptation of bacterial cells to their new environment following storage and thawing apparently slows down the metabolic activity of bio-cryogels to degrade phenol at early stages of incubation, without losing their activity completely or their ability to repair the freezing/storage damage as a result of adaptation processes.^46,47^ Assuming death of bacteria due to the freezing/thawing cycle, the mixture of bacterial strains showed less activity during the first week of incubation. During bioremediation the efficiency of the bioremediation process increases.

To explore the possibility of use of the material for other contaminants at comparable conditions a highly toxic and chemically stable 4-chlorophenol (4CP) was selected. It was shown that suspensions of bacteria did not degrade 4-chlorophenol in carbonate buffer, (Fig. S5a†) whereas cryogels based on *P. mendocina* slowly degraded chlorophenols (up to 40% degradation) over 24 days. There was no significant difference in bioremediation activity when different compositions of cross-linking agent were used (Fig. S5b†). Free suspensions of *P. mendocina* were more resistant to 50 mg L^−1^ of 4-chlorophenol compared to *Rh. koreensis* (Fig. S6†). It is noteworthy that bacteria previously adapted to phenol were not resistant to 50 mg L^−1^ of 4CP, and most of the bacteria died out within 24–28 h. *P. mendocina* adapted to 4CP on a plate revealed better survival compare to the non-adapted bacteria. *Rh. koreensis* in a suspension were not resistant to 4CP.

It should be noted that freezing temperatures may have a significant effect on the viability of cells. For instance,^48^ suggested that for long preservation periods (in excess of one year) −18 °C is a suitable freezing/preservation temperature for cells immobilised with PVA. In the current experiments, the samples have been frozen at −80 °C, which will reduce the percentage of live cells. The frozen samples needed more time to regain their efficiency to degrade phenol.

**Fig. 8:**
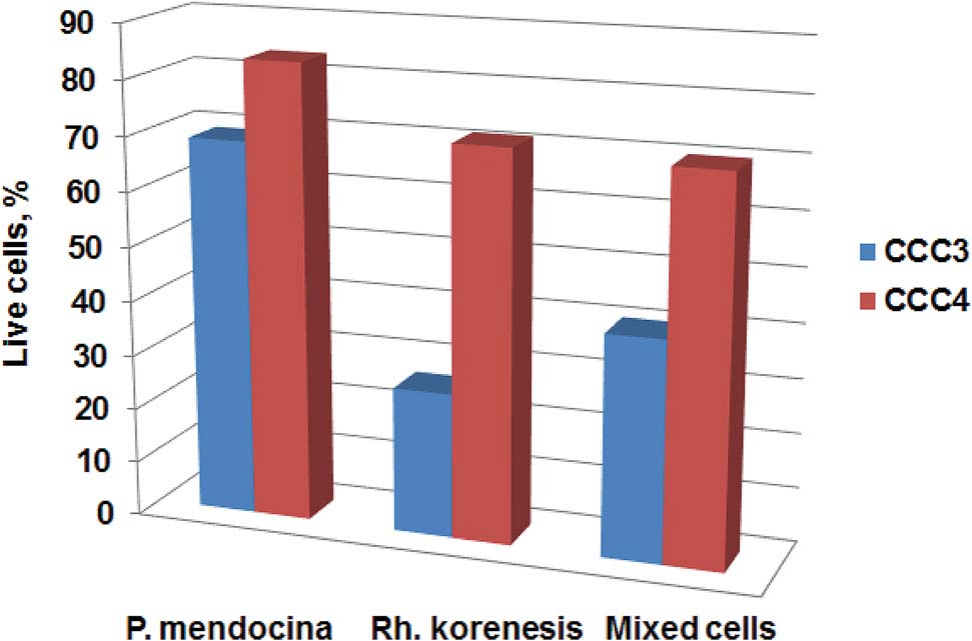
The viability of cells for CCC3 and CCC4 with *P. mendocina*, *Rh. koreensis* and their mixture (1 : 1 volume), after phenol degradation. Data are presented relative to the number of cells in a freshly prepared cryogel.

The viability of the bio-cryogels after exposure to phenol solution was also assessed using MTT assay after one incubation cycle. The results show a decrease in viability for CCC3 and CCC4 of *P. mendocina*, *Rh. koreensis* and their mixture (1 : 1) after incubation with phenol solution (Fig. 8). A decrease of 20–60% was observed for the CCC3 samples, greater than for CCC4 samples, where about 70–80% cells remained alive. Exposure of samples to phenol can significantly affect the viability of crosslinked cells. Previously it has been shown that phenol is a toxic compound and has harmful effects on living organisms even at low concentrations (1 mg L^−1^).^49,50^ In addition, the incubation media – carbonate buffer-may not provide sufficient nutrients for cell metabolism (which may, arguably, be less of a limitation in more concentrated waste waters containing a mix of organic and inorganic nutrients and micronutrients). However, the remaining percentages of live cells within bio-cryogels were able to degrade phenol for one week for samples crosslinked with (GA 0.5%, CCC1) and (PVA 1% + GA 0.5%, CCC2) following the collapse of the bio-cryogel structure. At the same time, the samples that were crosslinked with (PVA-al 1% + PEI-al 0.25%, CCC3) and (PVA-al 0.5% + PEI-al 0.6%, CCC4) were reused effectively for several cycles, even with a reduced percentage of live cells, which indicates that the remaining bacterial cells were adapted to the new environment.

## 4. Conclusion

A versatile and facile method for the preparation of 3D macroporous bacterial cell-based materials (bio-cryogels, or bacterial sponges) and their application for degradation of toxic compounds was demonstrated. The bacteria-based material was synthesized in a one-step process with a low percentage of cross-linking polymers and thus provides a high density of cells compared to other methods of cell immobilization. It was demonstrated that the complex porous bacterial construct could be made in one-step using more than one bacteria type. The method represents a more sustainable way to produce bioreactors based on bacterial cells due to the lower amount of waste generated after use and good potential biodegradability of the produced material. As we used a very low percentage of biocompatible polymer such materials could be simply composted and biodegraded. The cells were organised in a 3D porous structure with a well-developed system of interconnected pores (generating a 3D structured material) that provides efficient mass transport of nutrients and other products. Cross-linker systems based on PVA and PEI were designed and optimised so that a high percentage of cells remained viable, preserved their metabolic activity and continued to degrade phenol for five weeks while retaining their cross-linked cell structure. This degradative activity was maintained even after prolonged storage at −80 °C for 4–6 weeks demonstrating the possibility of long-term storage of the prepared materials and activation on demand, which is very important for transportation and industrial applications. In the current work we demonstrate only one application of such 3D structured bacteria based reactors. There are, however, a wide range of other potential applications such as reactors for the production of antibiotics, food supplements, and biosensors,^51^ and conversion of waste into bioenergy in microbial fuel cells.^51,52^

## Conflicts of interest

There are no conflicts of interest to declare.

## Supplementary Material

RA-008-C8RA04219E-s001
