# Supplementary material for: One-step formation of three-dimensional macroporous bacterial sponges as a novel approach for the preparation of bioreactors for bioremediation and green treatment of water
Source: RSC Adv. 2018 Sep 3;8(54):30813–24. doi: 10.1039/c8ra04219e (PMC9085471; doi:10.1039/c8ra04219e)
Supplement: RA-008-C8RA04219E-s001 [file RA-008-C8RA04219E-s001.pdf]

Electronic Supplementary Material (ESI) for RSC Advances.
This journal is © The Royal Society of Chemistry 2018

Supplementary information

One-step formation of three-dimensional macroporous bacteria biofilm sponge as a novel approach for preparation of bioreactor for bioremediation and green treatment of water.

Areej K.Al-Jwaid[1&2*], Dmitriy Berillo[3*], Irina Savina[3**], Andrew B.Cundy[4], Jonathan L.Caplin[1]

[1] School of Environment and Technology, University of Brighton, Brighton, UK.

[2] Engineering Technical College/ Basrah, Southern Technical University, Basrah, Iraq.

[3] School of Pharmacy and Biomolecular Sciences, University of Brighton, Brighton, UK.

[4] Ocean and Earth Science, University of Southampton, Southampton, UK.

*Both authors contributed equally to this manuscript.

**Corresponding author e-mail: i.n.savina@brighton.ac.uk

Graphical abstract

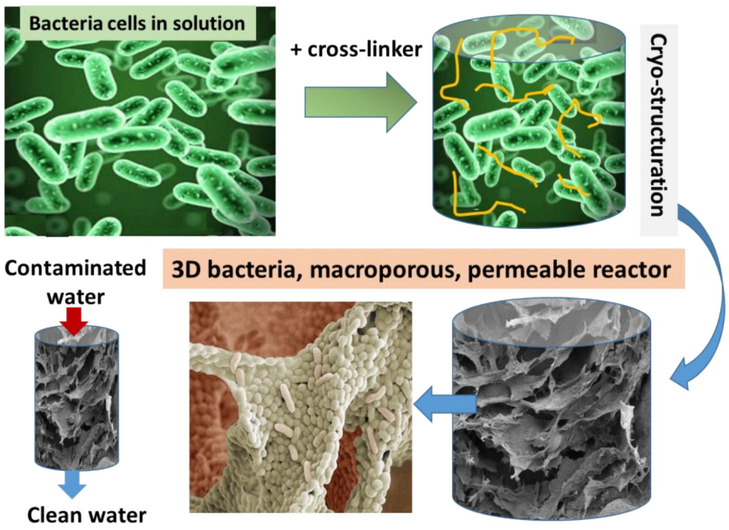

Polymer analysis.

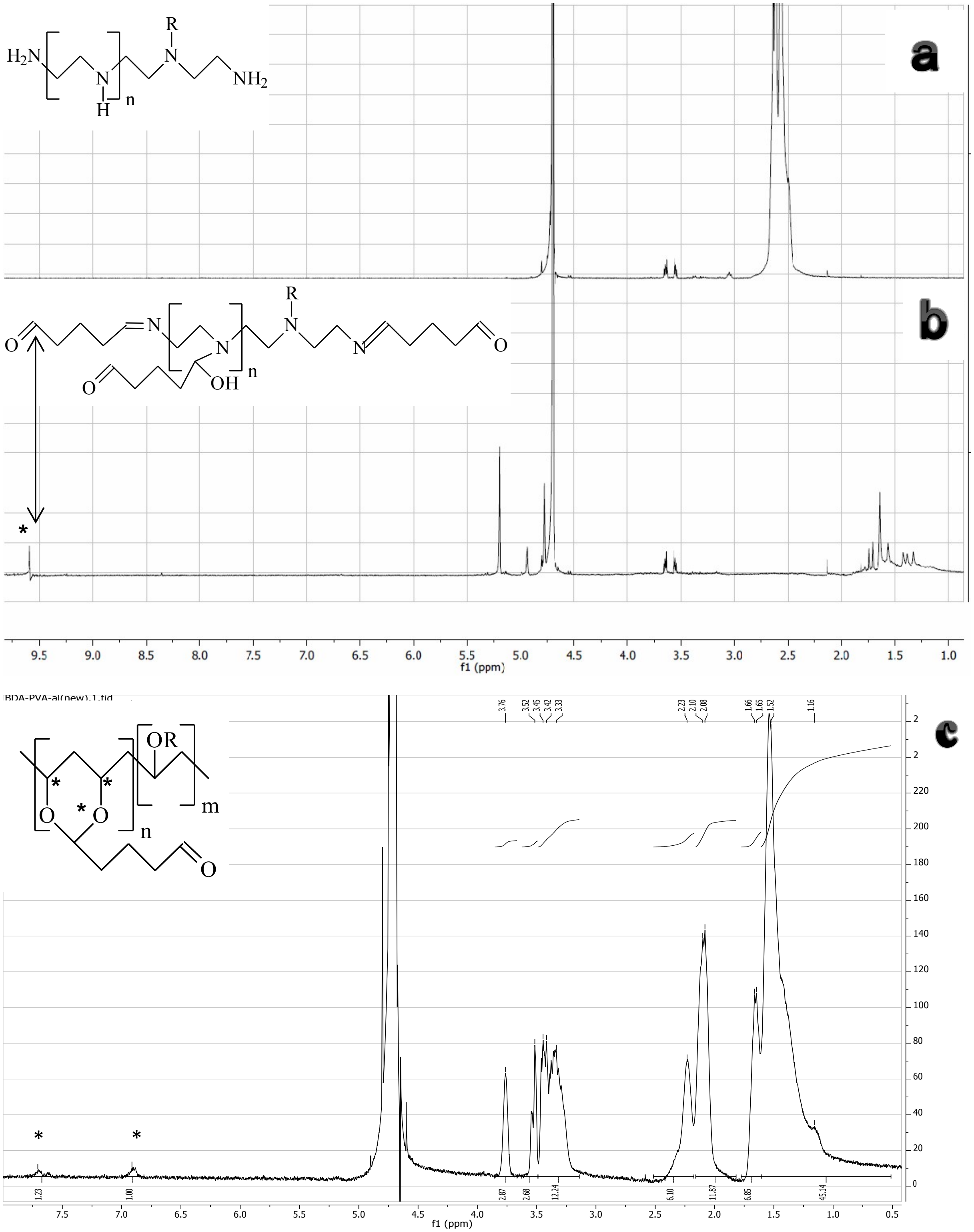
a
b
c
f1 (ppm)
BDA-PVA-al(new).1.fid
OR
OH
NH2
H2N

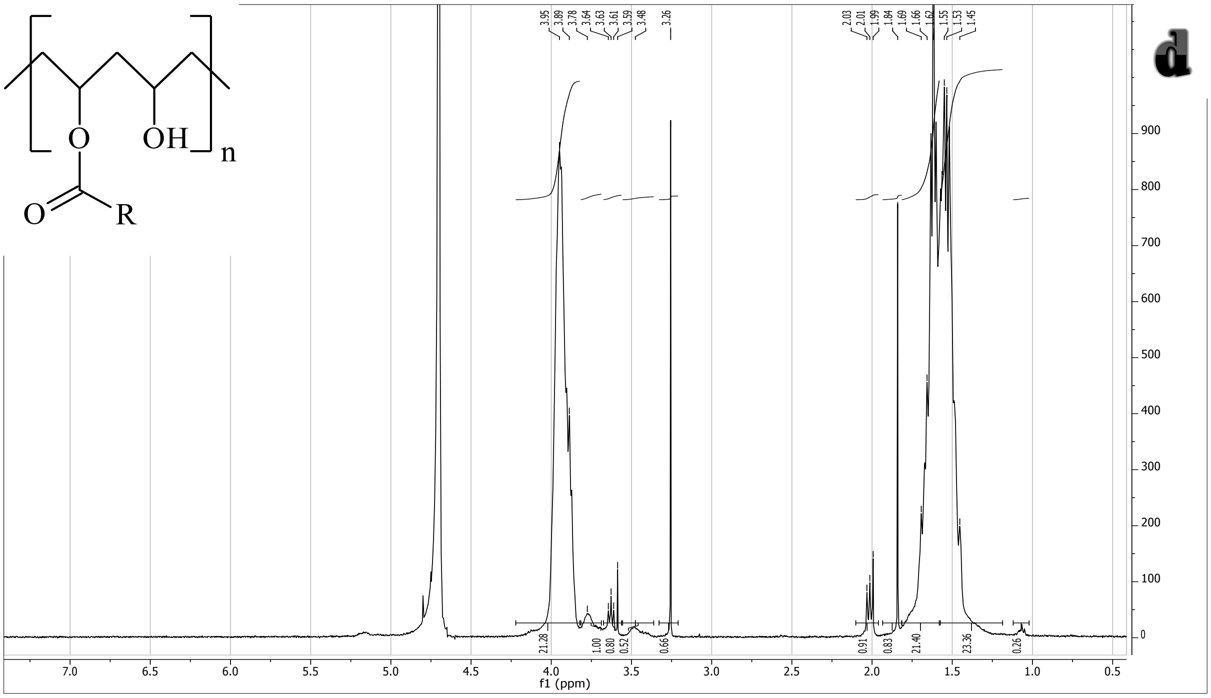

O
OH
n
O
R
d
3.95 3.89 3.78 3.64 3.63 3.61 3.59 3.48 3.26
2.03 2.01 1.99 1.84 1.69 1.66 1.62 1.55 1.53 1.45
21.28 1.00 0.80 0.52 0.66 0.91 0.83 21.40 23.36 0.26
7.0 6.5 6.0 5.5 5.0 4.5 4.0 3.5 3.0 2.5 2.0 1.5 1.0 0.5
f1 (ppm)
900 800 700 600 500 400 300 200 100 0

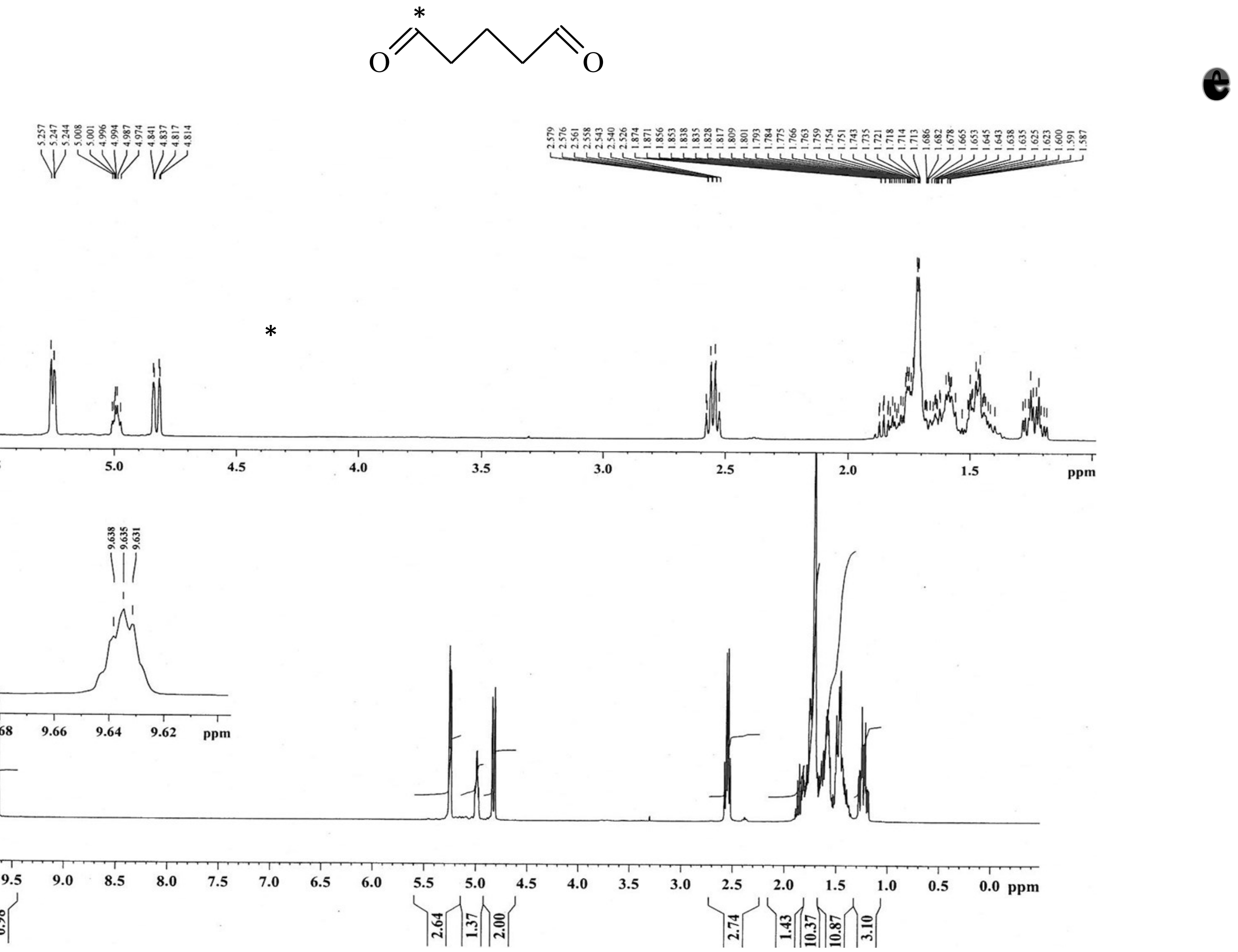

Fig. S1. H-NMR-spectra of a) PEI, b) PEI-al, c) PVA-al, d) PVA, e) glutaraldehyde in D_2O.

B

-C(=O)**H**

↓

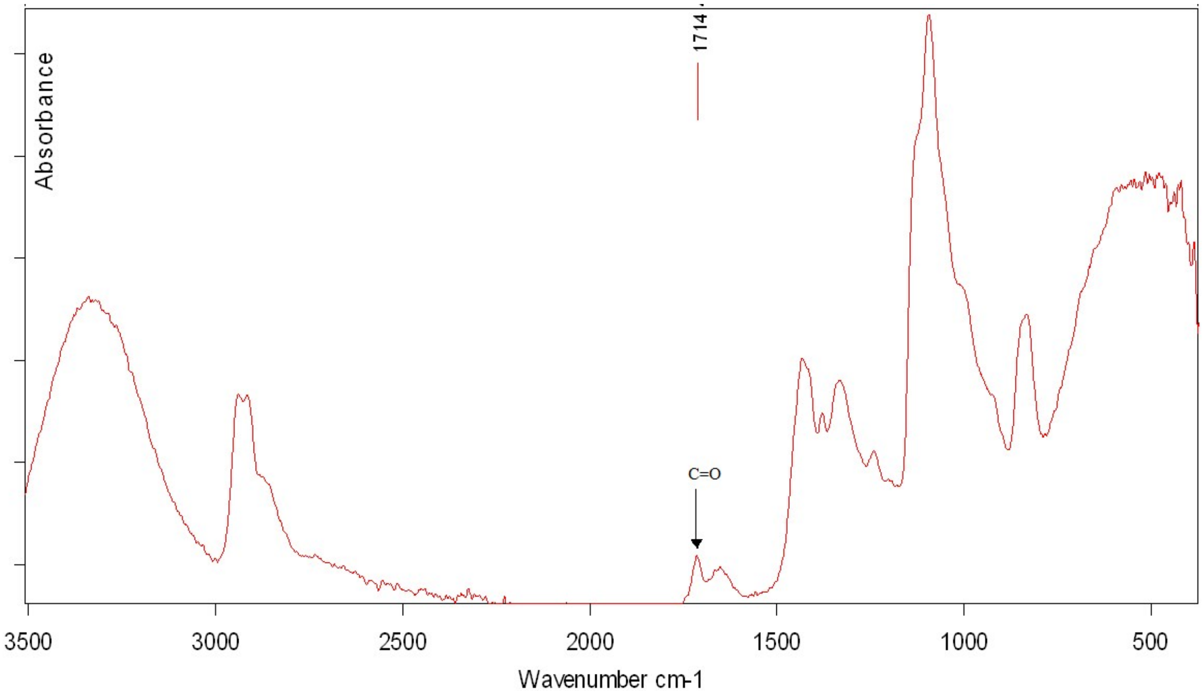

Figure S2. FTIR-spectrum of PVA-al

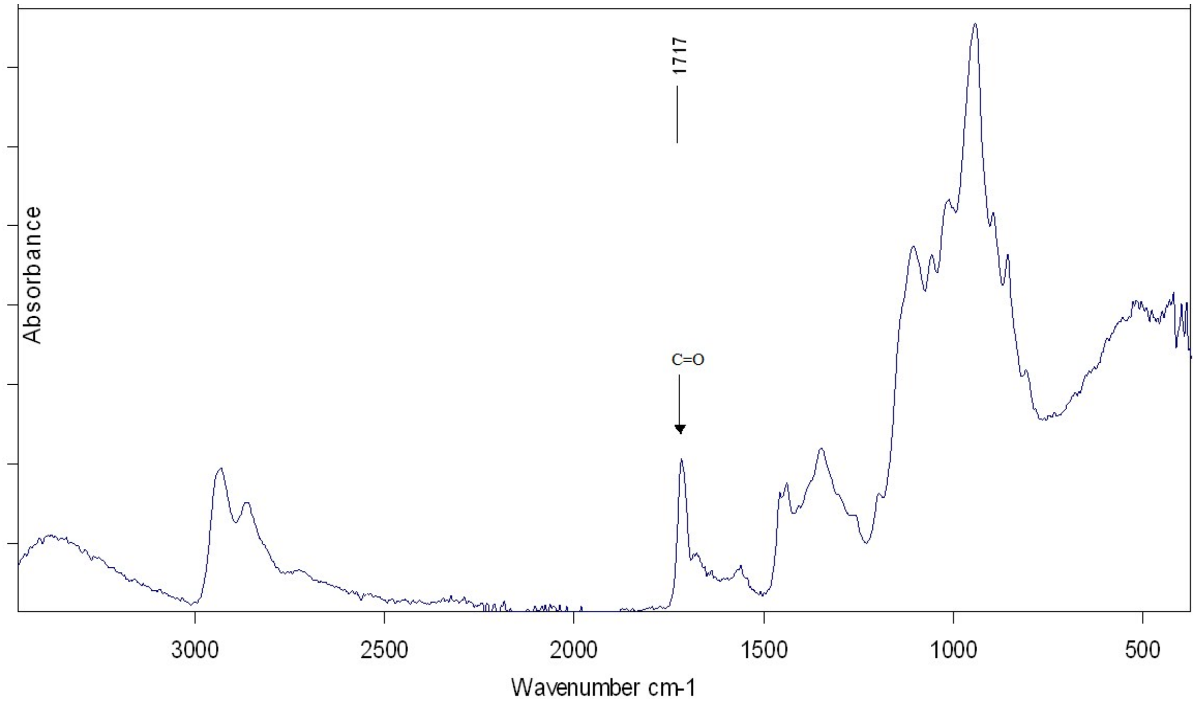

Figure S3. FTIR-spectrum of PEI-al.

HPLC settings and analysis.

Mobile phase 1% v/v acetic acid in water (C) and 1% v/v acetic acid in methanol (D). The method details: column temperature 45C, sample temperature 25C, C) Gradient Start at 75.0, End at 44.0%, D) Gradient Start at 25.0, End at 56.0% for 9.5minutes, and at composition 44%-56% C-D for 5min. C) Gradient Start at 44.0, End at 0.0% with duration of 1.0 min and D) Gradient Start at 56.0%, End at 100.0%, for 1.0min. 5 minutes at 100% of CH3OH&1% HAc. C) Gradient Start at 0.0, End at 75.0% for 1.0min; D) Gradient Start at 100.0, End at 25.0% with duration of 1.0min.

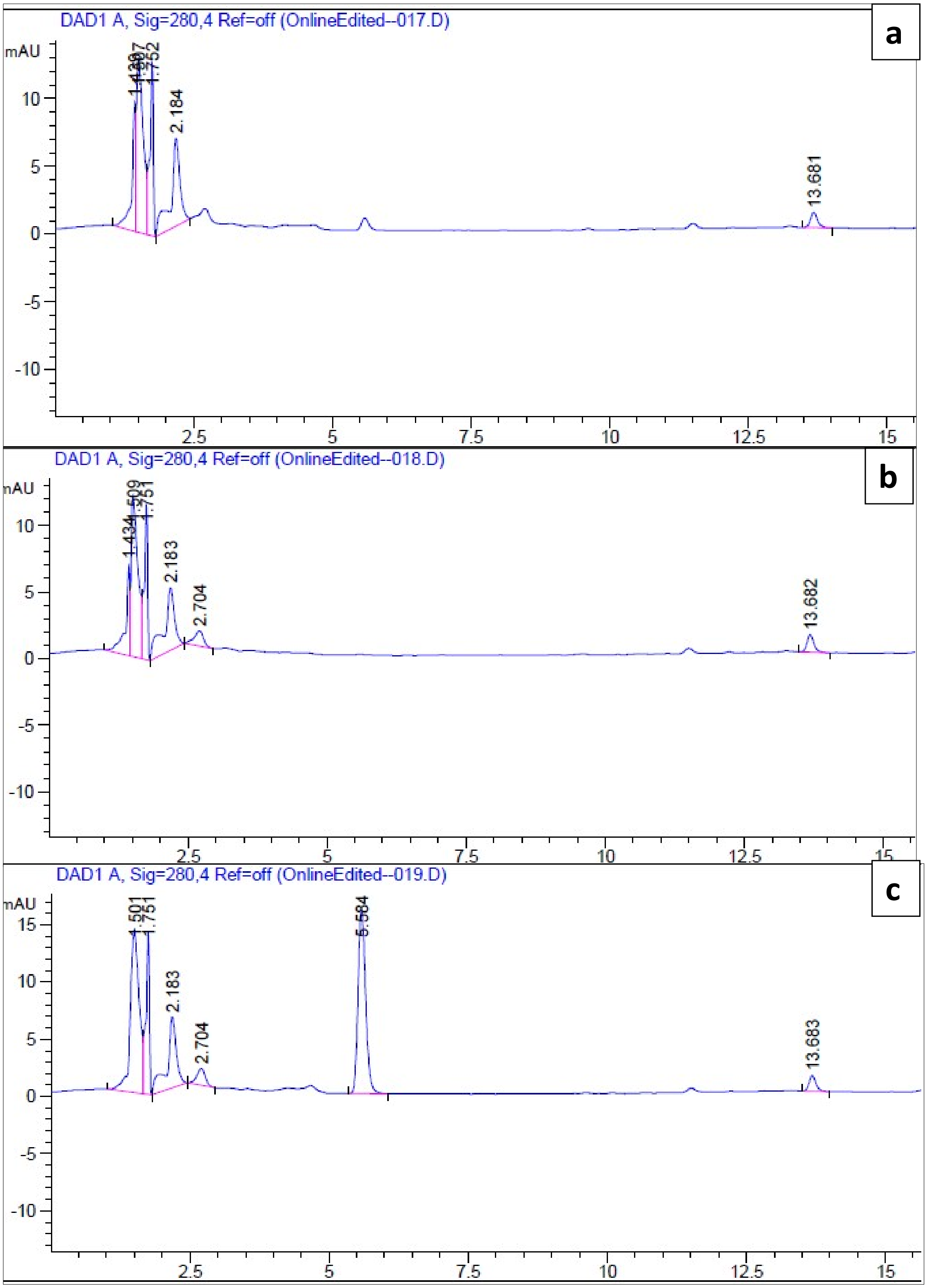

Figure S4. HPLC chromatogram of the solution after phenol degradation by the cryogel composed of: a) mixed bacteria strains *Rhodococcus koreensis and Pseudomonas mendocina;* b) *Rhodococcus koreensis c) Pseudomonas mendocina*.

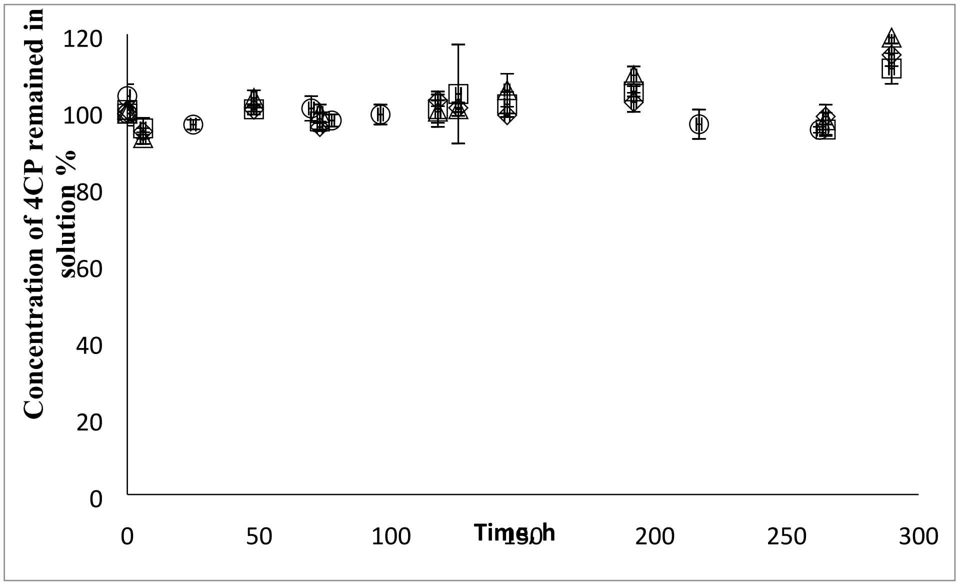

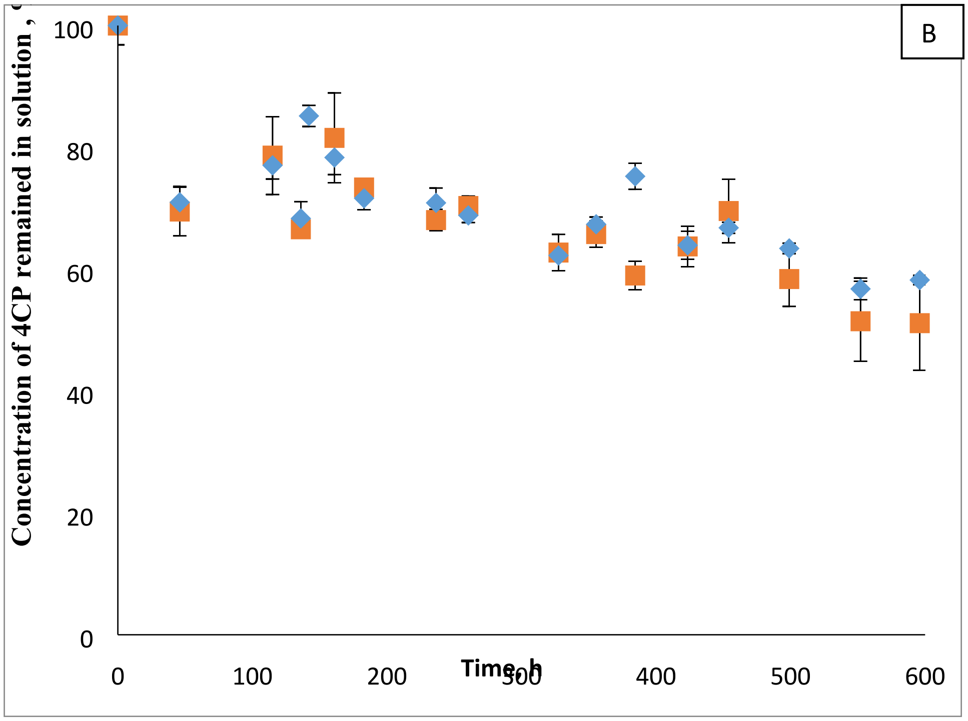

Figure S5. Degradation kinetic of 4CP (V 40ml 50mg/L) in 25mM carbonate buffer**,** dynamic mode (shaking at 150 rpm): A) suspension of free *Rhodococcus koreensis* commercial(triangle); *Rhodococcus koreensis* 4CP adapted(square); *Pseudomonas mendocina* commercial(circle) *Pseudomonas mendocina* 4CP adapted number of cell $0.7x10^8$ per sample (n=3). B) Cryogels Pseudomonas PVA-al 1.7% (square), Pseudomonas PVA-al –PEI-al 0.6-0.5 % (rhomb).

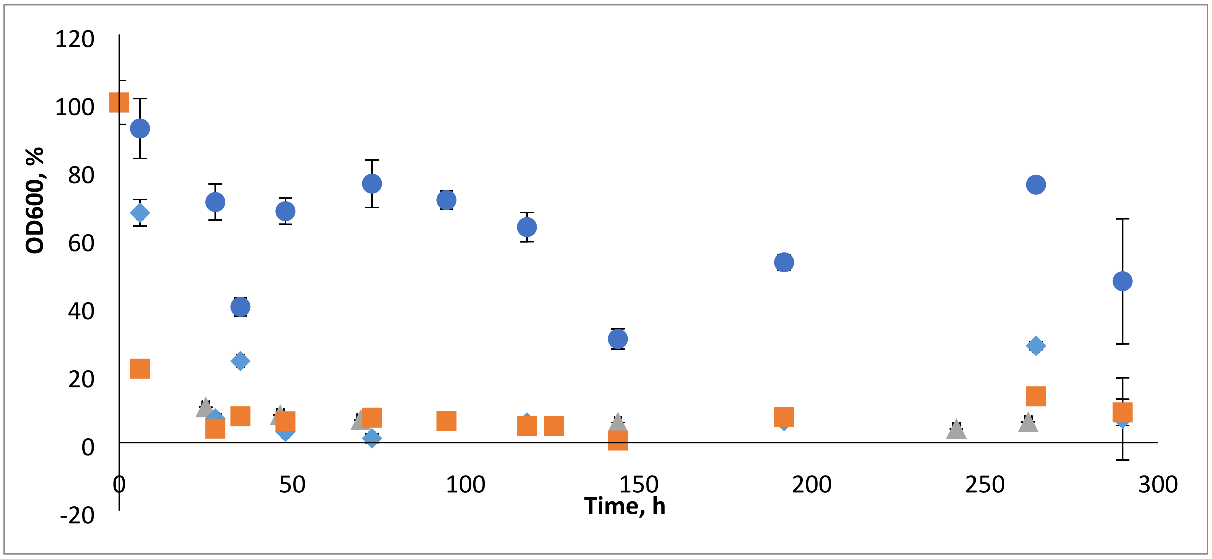

Figure S6. Kinetic of change of viable bacteria in the 50 mg/L of 4CP solution in 25 mM carbonate buffer in a dynamic mode (shaking at 150 rpm): *Pseudomonas mendocina* adapted to phenol (triangle); *Pseudomonas mendocina* commercial (circle); *Rhodococcus koreensis* (square) and *Rhodococcus koreensis adapted to phenol(rhomb)*.
